# Predictive modeling with linear machine learning can estimate glioblastoma survival in months based solely on MGMT-methylation status, age and sex

**DOI:** 10.1007/s00701-025-06441-7

**Published:** 2025-02-24

**Authors:** Emanuele Maragno, Sarah Ricchizzi, Nils Ralf Winter, Sönke Josua Hellwig, Walter Stummer, Tim Hahn, Markus Holling

**Affiliations:** 1 Department of Neurosurgery, University Hospital Münster, Albert-Schweitzer-Campus 1 A, 48149 Münster, Germany; 2 Institute for Translational Psychiatry, University of Münster, Münster, Germany

**Keywords:** Machine learning framework, Glioblastoma multiforme, MGMT-methylation, Prognosis, Regression model

## Abstract

**Purpose:**

Machine Learning (ML) has become an essential tool for analyzing biomedical data, facilitating the prediction of treatment outcomes and patient survival. However, the effectiveness of ML models heavily relies on both the choice of algorithms and the quality of the input data. In this study, we aimed to develop a novel predictive model to estimate individual survival for patients diagnosed with glioblastoma (GBM), focusing on key variables such as O6-Methylguanine-DNA Methyltransferase (MGMT) methylation status, age, and sex.

**Methods:**

To identify the optimal approach, we utilized retrospective data from 218 patients treated at our brain tumor center. The performance of the ML models was evaluated within repeated tenfold regression. The pipeline comprised five regression estimators, including both linear and non-linear algorithms. Permutation feature importance highlighted the feature with the most significant impact on the model. Statistical significance was assessed using a permutation test procedure.

**Results:**

The best machine learning algorithm achieved a mean absolute error (MAE) of 12.65 (SD = ± 2.18) and an explained variance (EV) of 7% (SD = ± 1.8%) with *p* < 0.001. Linear algorithms led to more accurate predictions than non-linear estimators. Feature importance testing indicated that age and positive MGMT-methylation influenced the predictions the most.

**Conclusion:**

In summary, here we provide a novel approach allowing to predict GBM patient’s survival in months solely based on key parameters such as age, sex and MGMT-methylation status and underscores MGMT-methylation status as key prognostic factor for GBM patients survival.

## Background

Glioblastoma multiforme (GBM) are the most common primary malignant brain tumors in adults [30, 32, 38], occurring with an incidence of approximately 3 cases per 100,000 persons [31, 32]. Despite maximal safe surgical resection, and postoperative concurrent radiochemotherapy, GBM patients show a poor prognosis with a two-year survival rate of 27% and 5-year survival rate of 5% [31].

In recent years, molecular diagnostics gained substantial clinical relevance serving as prognostic marker that influence therapeutic decision-making [7, 27, 35]. A critical genetic alteration with significant implications for therapeutic strategy is the methylation of the MGMT promotor [14, 29, 45]. The MGMT gene resides on chromosome 10 (10q26) and encodes for a DNA-repair-enzyme O- 6-methylguanine-DNA-methyltrasferase that removes alkyl groups from the O6 position of guanine, a site frequently targeted by alkylating agents like temozolomide [2, 37]. When the MGMT promotor is methylated, the expression of the MGMT enzyme is silenced, reducing the tumor’s ability to repair the DNA damage. Stupp et al. [40] and others [11, 18, 44] demonstrate a positive correlation between silenced MGMT-promotor, progression-free survival (PFS) and overall survival (OS) following treatment with the alkylating agent temozolomide. The Nordic Elderly trial [18] and the NOA 08 study [44], show a significantly longer survival in elderly patients (> 60y and > 65y) with TMZ alone and MGMT promoter methylation, compared with TMZ with unmethylated tumors. The EANO 2021 guidelines on diagnosing and treating diffuse gliomas in adults underline again the importance of methylation in guiding treatment decisions on chemotherapy with alkylating agents [43].

For this reason, further analysis on the impact of different MGMT-methylation status is of interest. The clinical implications of inconsistently methylated MGMT promotors remain unclear between truly methylated and non-methylated tumors, which best fit the defined prognostic model. This condition applies to tumors where methylation peaks are detected in some but not all PCR replicates.

In recent years, machine learning gains significant prominence in tumor research, including studies on GBM.

Machine learning allows for the prediction of individual outcomes, as opposed to values that would otherwise pertain to the mean or median of a population. When considering the use of machine learning as a prognostic tool for patient survival—especially based on clinical parameters like treatment regimen, MGMT methylation status, and the inclusion of patients with tumor progression or recurrences—the development and implementation of an effective machine learning model remains a significant challenge [20, 33]. Therefore, the aim of this study was to evaluate a new ML model that may allow more precise prediction of patient’s survival compared to classical univariate models [24]. With our study, we aim to develop a regression model capable of providing individualized prognostic predictions for patients with GBM. Unlike classification models that divide patients into subgroups, our approach seeks to predict individual survival in months. To ensure ease of use in clinical practice, we have included the most relevant clinical variables, with a particular focus on the influence of MGMT methylation. Specifically, we aim to evaluate how MGMT methylation, categorized as positive, intermediate, or negative, carries different prognostic weights.

## Methods

### Study population

We enrolled 253 adult patients in this study, consecutively operated between 2015 and 2018 in our institute with a diagnosis of glioblastoma multiforme. Clinical variables were age at baseline, sex (female (f)/male (m)), MGMT methylation (positive, intermediate), and type of therapy (STUPP-Protocol: combined overall 60 Gy radiotherapy and temozolomide 75 mg per square meter body surface for six weeks, followed by six cycles of temozolomide alone/Definite Radiotherapy. We divided MGMT methylation into 3 clinical variables depending on the intensity of methylation: methylation positive, intermediate methylation positive, and no methylation. For therapy, we used patients who underwent Stupp-Protocol and definite radiotherapy. We extrapolated this information from our electronic medical records and partially through phone interviews. With these variables, we aim to predict overall survival in months.

### Inclusion criteria

To be enrolled in our data set, the patients must undergo a glioma operation in our neurosurgical department between 2015 and 2018, with corresponding histological and molecular characterization, IDH-wildtype, including methylation status and a pathology diagnosis resulting in WHO 4 (2021, Classification). By secondary malignant neoplasm, we registered the time of malignization of the histological results. This resulted in 253 patients in total. We also included only those patients for whom we had complete information on all variables to avoid inaccuracies due to imputations. Our final number for our most extensive dataset resulted in 218 patients.

### Histopathological analysis

We mainly focused on the difference in the clinical prognosis of truly methylated, not methylated, and inconsistently methylated tumors. DNA was extracted from each histological probe and analyzed in our institute using a MS-PCR to set the three groups. Duplicate bisulfite reactions were performed for each DNA sample, followed by duplicate PCRs. After that, a capillary electrophoresis was performed, and the methylation picks were observed. According to the band concentration, our Institute of Pathology defined the methylation status based on the concentration of the methylation bands. A tumor with MS-band concentration < 0,1 ng/μL was characterized as not methylated. A MS-band concentration > 0,5 ng/μL was defined as methylated. A concentration between 0,1 and 0,5 ng/μL was characteristic for an inconsistently methylated tumor.

### Statistical analysis

Focusing on MGMT-methylation as the main factor for our research question, we conducted a univariate statistical analysis using an Analysis of Variance (ANOVA) model in IBM SPSS (Version 29.0.2). In this analysis, MGMT-methylation was treated as a three-factor model, and age and sex were included as covariates to predict overall survival. We considered p-values less than 0.05 to be statistically significant.

### Machine learning model

For our machine-learning analysis, we used the programming language Python 3.8 on PHOTONAI [23, 24], a machine-learning platform for implementing and visualizing machine-learning models.

We implemented five different algorithms for prediction; of which three were linear and two non-linear models: Linear Regression, linear Support Vector Regression (SVR), non-linear SVR, Random Forest, K-nearest Neighbours.

Linear Regression fits a line to data by minimizing squared errors to model the relationship between variables, while linear Support Vector Regression finds a line within a specified tolerance to maximize margins and effectively handles both linear and non-linear relationships. Random Forest Regression averages predictions from multiple decision trees to capture complex relationships and is robust against overfitting, while the K-Nearest Neighbour (KNN) algorithm predicts target values based on the average or most frequent values of the nearest neighbors.

For Hyperparameter optimization we integrated a nested 10 by tenfold regression with 5 repeats to optimize hyperparameters of the models and estimate the out-of-sample prediction performance. To evaluate the models, we used mean squared error (MSE), mean absolute error (MAE), explained variance (EV), Pearson correlation (PC), and R-squared (r^2^). We calculated the performance on a null information estimator as a reference model. This reference model predicts the mean survival rate of the individuals in the training set.

To determine the influence of MGMT-methylation, we added a feature importance analysis to identify the variables with the highest impact on the predictive model. Permutation feature importance measures the impact of each feature by evaluating the model's performance with shuffled values of that feature. The procedure involves randomly shuffling the values of one feature in the dataset, making predictions with the modified dataset, and calculating the decrease in model performance (MAE). This decrease in performance indicates the importance score. To statistically evaluate if the machine learning model performs better than random chance, we conducted a permutation test. In a permutation test, we began by shuffling the target variable while keeping the features unchanged. We then evaluated the model's performance on this shuffled dataset and repeated the process multiple times to generate a distribution of performance metrics under the null hypothesis. Finally, we compared the model's performance on the original dataset to this distribution to determine if the observed performance is statistically significant.

## Results

### Study population

Retrospective data from 218 patients (123 male, 95 female) that underwent treatment at our brain tumor center were included. The patient’s age ranged between 25 and 84 years with a median of 64 years (± 11.6 SD). 95.4% of the patients were treated according to the Stupp protocol (citation) and 4.6% received radiotherapy only. Overall survival of all patients ranged from 0–101 months with a median survival of 21 months (± 17.9 SD, Table 1).

**Table 1:** Population overview

Variable	Population (*n* = 218)
Age	
Total	25–84 years
Median	64 years, ± 11.6 SD
Sex	
Female	43.6% (*N* = 95)
Male	56.4% (*N* = 123)
Adjuvant Therapy	
Stupp-Protocol	95.4% (*N* = 208)
Definite Radiotherapy	4.6% (*N* = 10)
MGMT-Methylation	
Positive	42.7% (*N* = 93)
Intermediate	19.7% (*N* = 43)
Negative	37.6% (*N* = 82)
Overall Survival	
Total	0–101 months
Median	21 months, ± 17.9 SD

Overall, the median age distribution is 64 years, with men being more frequently affected, showing a majority of positive MGMT methylation, and predominantly undergoing therapeutic application of the STUPP protocol. The overall survival demonstrates a median of 21 months

### Statistical analysis

We calculated an ANOVA model to investigate the association of MGMT-methylation and overall survival. We included age and sex as covariates in the ANOVA model. MGMT was significantly associated with overall survival (F(2,213) = 3.6, *p* = 0.029).

### Machine learning analysis

**Fig. 1:**
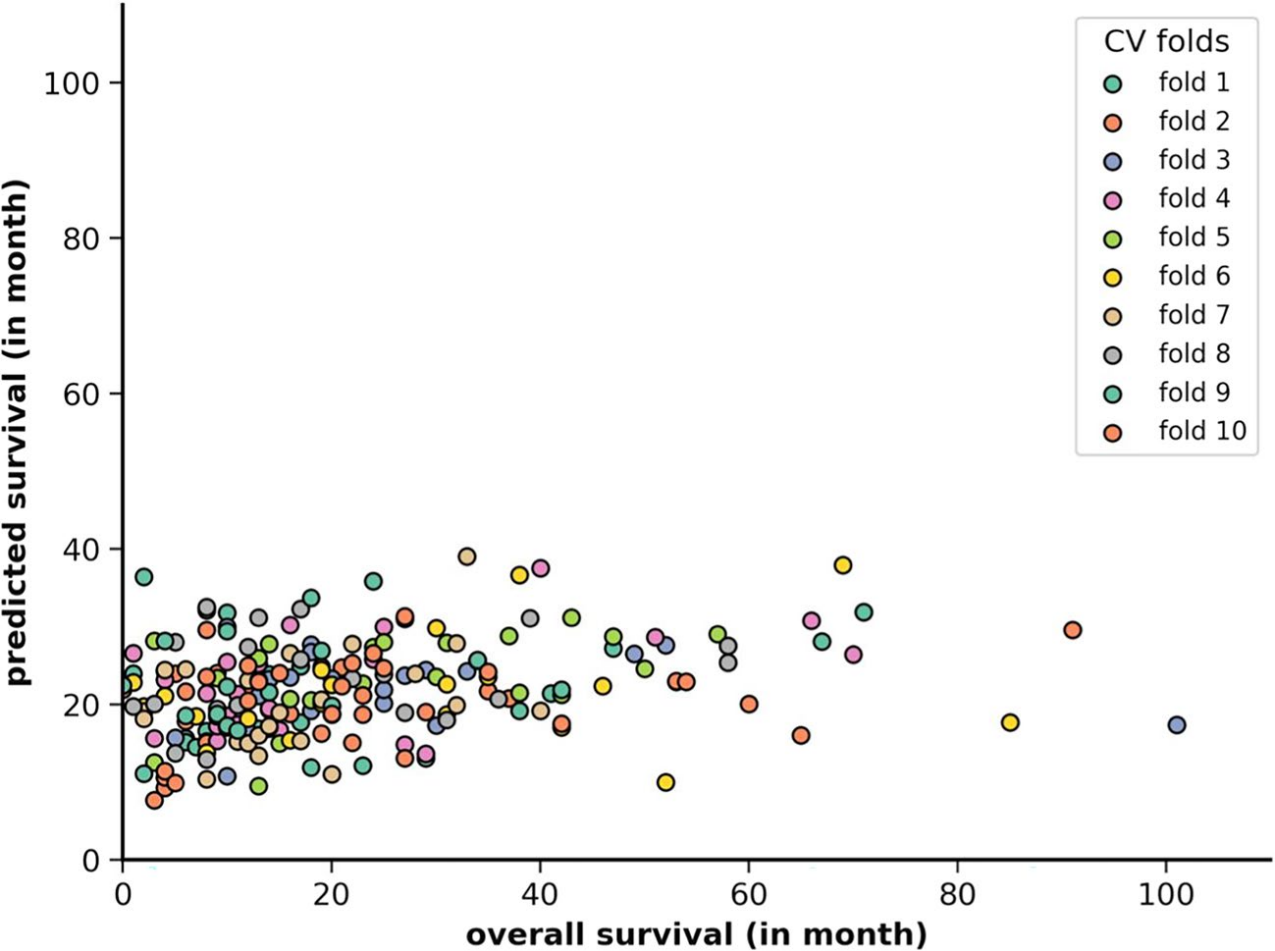
Visualization of predictions: X-axis shows the data of true overall survival in months, y-axis shows the predicted survival in months. The diagram shows overall a linear direction of all predictions. The 10 folds represent the dataset divided into 10 sets. Each point represents the color of the given fold

Overall, Linear Regression turned out to be the best algorithm for prediction, resulting in a near-linear predictive visualization (MAE = 12.65, MSE = 296.26, Table 2). Linear Regression also showed better results compared to Null Information Rate (MAE = 13.65, MSE = 323.39), indicating the predictive stability of our model and prove of concept. The visualization of the dataset highlighted the nearly linear prediction of our data. This further confirmed that a linear model achieved the best results, as the distribution of our data follows a linear pattern (Fig. 1).

**Table 2:** Overview comparison of all algorithms

Algorithm Name	MSE	MAE	EV	PC	R2
Null Information Rate	323.39	13.65	0.00	NA	− 0.065
Linear Regression	296.26	12.65	0.07	0.31	0.03
Linear SVR	364.96	13.13	0.01	0.11	− 0.19
SVR	310.20	12.18	0.08	0.31	− 0.01
Random Forest Regressor	327.45	13.35	− 0.04	0.18	− 0.10
K Neighbour Regressor	352.88	14.06	− 0.14	0.15	− 0.20

Results of all used algorithms (Linear Regression, Linear SVR, SVR, Random Forest Regressor, K Neighbour Regressor) differentiated by the metrics MSE (Mean Squared Error), MAE (Mean Absolute Error), EV (Explained Variance), PC (Pearson Correlation) and R2 (R-Squared). In addition to the algorithms, the Null Information Rate serves as a reference. Linear Regression is the best model on the pipeline with MAE of 12.65 and EV of 7%, compared to K Neighbour Regressor (MAE = 14.06, EV = − 14%), which showed worse results than the Null Information Rate (MAE = 13.65, EV = 0%). Overall, linear algorithms show better results compared to the non-linear estimators: Linear Regression (MAE = 12.65), Linear SVR (MAE = 13.13), and SVR (MAE = 12.18), all showing better results than Null Information Rate

**Fig. 2:**
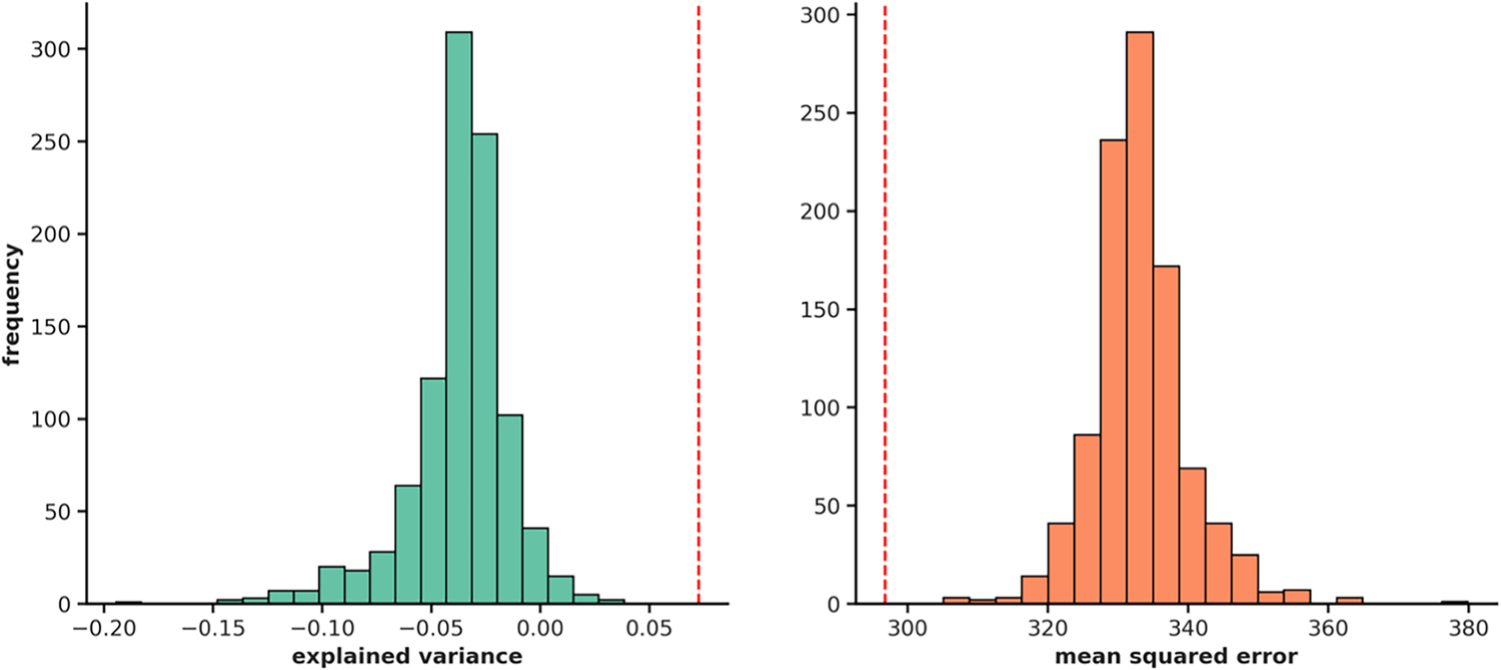
Permutation testing: Repeat of 1000 iterations to validate the strength of our model. The left diagram shows the results of the metric Explained Variance, the X-axis showing the true results of each metric, the Y-axis showing the number of repeats (frequency). The left diagram shows the results of the metric Mean Squared Error, the X-axis showing the true numbers of all results of this metric, the Y-axis showing the number of repeats. The red line on each diagram represents the test statistic of our original metrics, showing significant results through permutation testing

In the permutation test, we proved a general stability of the model (Fig. 2) and thus showed the significance of the results (*p* < 0.001).

Finally, the non-linear models were identified as the least effective algorithms for this dataset: Random Forest Regressor with MAE of 13.35, MSE of 327.45, EV of − 0.14 and K Neighbour Regressor with MAE of 14.06, MSE of 352.88 and EV of − 0.14 (Table 2).

**Fig. 3:**
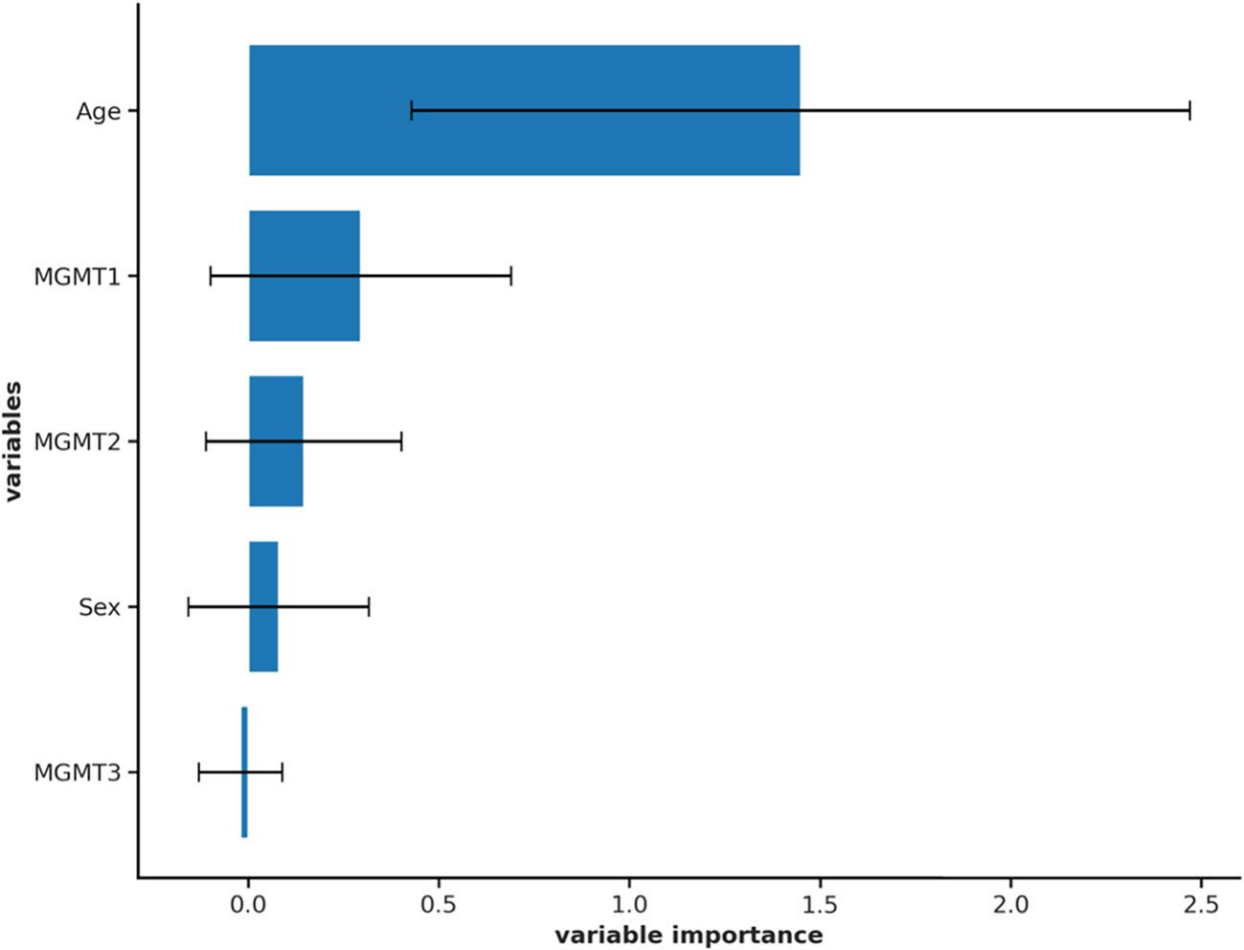
Feature Importance: X-axis showing the variable importance of each variable, y-axis showing the different variables, best being age, followed by positive MGMT-methylation (MGMT1), intermediate MGMT-methylation (MGMT2), sex and no MGMT-methylation (MGMT3)

Feature importance showed that age had the greatest influences on the prediction (importance (mean) 1.45 SD ± 1.02, Fig. 3). Regarding MGMT methylation, positive MGMT methylation had the highest impact (importance (mean) = 0.29 SD ± 0.39), while no MGMT methylation had the least (Table 3). It also emphasized that intermediate MGMT-methylation (MGMT 2, Table 3) had little to no influence on the model (Importance mean = 0.14, Rank = 3).

**Table 3:** Feature importance

Metric	Age	Sex	MGMT 1	MGMT 2	MGMT 3
Importance (mean)	1.45	0.08	0.29	0.14	− 0.02
Importance (SD)	1.02	0.24	0.39	0.26	0.11
Importance (rank)	1	4	2	3	5

The table shows an overview of the feature importance for the five features used. Age has the highest influence (importance (mean) = 1.45), followed by positive MGMT methylation (MGMT 1, importance (mean) = 0.29) and intermediate positive MGMT methylation (MGMT 2, importance (mean) = 0.14). Sex and no MGMT methylation (MGMT 3) shows the least influence.

## Discussion

Machine learning offers promising avenues to assess biomedical data related to patient’s disease course and survival. Our study demonstrates that an effective machine learning model can be constructed within a multivariate framework, even when limited to three clinical parameters. Importantly, our data confirm the influence of established parameters, such as the patient’s age at diagnosis and MGMT-methylation status, on overall survival and prognosis. Despite utilizing a limited number of variables, our model outperformed the null hypothesis model. It provides a solid foundation for further research into small-scale machine learning models aimed at enhancing clinical applicability.

Previous studies predominantly focus on classification models based on MRI data [34] categorizing outcomes into short- and long-term survival [21, 26] or employing regression models without clinical features [39]. While these methods highlight group-level differences, they fail to provide individualized predictions. Our approach seeks to bridge this gap, although further refinement is needed to enhance accuracy and applicability.

In our study, the linear model outperformes the non-linear approach, likely due to the limited number of variables involved. This finding aligns with previous research showing mixed results when comparing Cox regression with Random Forest and SVM [8, 36]. While some studies show Cox regression superior [36] others favor Random Forest [8]. Predictions for clinical outcomes, particularly usw medical imaging [6, 28] and next-generation sequencing [17]. Currently, a model comparing Random Forest and linear statistics has been tested and concludes that Random Forest results in accurate predictions. However, the model used more parameters and a larger patient population [20].

Our results suggest that more complex models could offer greater accuracy, mainly when dealing with heterogeneous patient cohorts and diverse clinical parameters. In comparison, a model with standard statistical analysis [12] has been performed to estimate survival.

Developing robust machine learning models for clinical applications faces several challenges. The heterogeneity of patient cohorts and the rarity of certain diseases complicate model development. Additionally, variability in how clinical parameters are evaluated across different clinics hampers comparability and external validation [10, 41]. While machine learning continues to gain traction, issues such as overfitting and the interpretation of model outputs remain significant concerns [19].

The next challenge is understanding the offered results of a machine learning model [1]. Other challenges that have affected us include the wide range of clinical parameters and the reduction and capture of the most essential parameters to be used in the model. Effective model development requires interdisciplinary collaboration from the data collection phase to ensure the capture of relevant data points.

Future efforts should focus on prospective models developed through close collaboration among hospitals, pathology centers, and data scientists to ensure larger patient cohorts and improve model comparability [9].

Our model confirms the prognostic influence of MGMT-methylation status. However, it did not show a significant impact of the intermediate state of MGMT-methylation regarding the prognostic value on overall survival. Through our analysis, we were able to confirm the impact of positive MGMT methylation on prognosis, as already demonstrated by previous studies. Similarly, we showed that the absence of MGMT methylation (negative MGMT methylation) has little impact on prognosis. The intermediate state of MGMT-methylation has emerged as a significant variable under the new WHO Classification of 2021 [13]. A few studies started looking deeper into the impact of intermediate state MGMT-methylation [3, 16, 25]. One study framed the intermediate state to be a grey zone in which a prognostic value could be possible [25]. With this we could emphasize future studies should investigate the impact of this intermediate state on prognosis and therapeutic approaches.

### Limitation

A notable limitation of our study is the retrospective nature of data collection, introducing potential biases. The sample size may also affect the generalizability of our findings. Furthermore, the contentious methods and cut-off values for determining MGMT status present challenges [43]. Nevertheless, it remains discussed which analytic method should be used as standard. Furthermore, quite apart from the used technique, it seems to prevail a relatively high discordance in the MGMT results in central or local tests (about 39% [22]). Various studies and meta-analyses also found that the IHC method is not in close concordance with MSP-PCR analysis [4], nor with Pyrosequences [42]. Moreover, discussion continues regarding the cut-off value for defining whether an MGMT-Promoter is methylated or not [5, 15, 46, 47]. Due to the poorly comparable values of MGMT methylation, we decided against testing our analysis externally using a dataset from another clinic. Our evaluation specifically focused on intermediate MGMT methylation. Since this does not have a defined value in molecular diagnostics, it would represent a different variable from the one we are using. Therefore, conducting an external validation would not be effective for ensuring data comparability.

Overfitting remains a concern, particularly in high-grade gliomas and heterogeneous patient populations. Further validation in additional prospective cohorts is needed to confirm our model’s robustness and clinical utility.

## Conclusion

Our study outlines challenges and limitations that affect the development of predictive models and indicates the potential of predictive models utilizing linear machine learning techniques to estimate overall survival in glioblastoma patients, based on a small set of key variables such as the MGMT methylation status. We could confirm the impact of positive MGMT-methylation on the prognosis of GBM patient, whereas the intermediate state MGMT methylation showed little influence on our model.

## Data Availability

No datasets were generated or analysed during the current study.

## Abbreviations

CNS: Central nervous System
EV: Explained variance
GBM: Glioblastoma multiforme
HGG: High Grade Gliomas
MAE: Mean absolute error
MGMT: Central O6-methylguanine-DNA methyltransferase
MSE: Mean squared error
PC: Pearson correlation
PFS: Progression Free Survival
PCR: Polymerase-Chain-Reaction
OS: Overall survival
r^2^: R-squared
SD: Standard Deviation

## References

[1] Adlung L, Cohen Y, Mor U, Elinav E (2021) Machine learning in clinical decision making. Med (N Y) 2:642–665. 10.1016/j.medj.2021.04.006 10.1016/j.medj.2021.04.006 35590138

[2] Bady P, Delorenzi M, Hegi ME (2016) Sensitivity analysis of the MGMT-STP27 model and impact of genetic and epigenetic context to predict the MGMT methylation status in gliomas and other tumors. J Mol Diagn 18:350–361. 10.1016/j.jmoldx.2015.11.009 26927331 10.1016/j.jmoldx.2015.11.009

[3] Bomsztyk K, Mar D, Denisenko O, Powell S, Vishnoi M, Yin Z, Delegard J, Hadley C, Tandon N, Patel AJ et al (2024) Analysis of DNA methylation in gliomas: assessment of preanalytical variables. Lab Invest 104:102160. 10.1016/j.labinv.2024.102160 39426568 10.1016/j.labinv.2024.102160 PMC11709230

[4] Brell M, Ibáñez J, Tortosa A (2011) O6-Methylguanine-DNA methyltransferase protein expression by immunohistochemistry in brain and non-brain systemic tumours: systematic review and meta-analysis of correlation with methylation-specific polymerase chain reaction. BMC Cancer 11:35. 10.1186/1471-2407-11-35 21269507 10.1186/1471-2407-11-35 PMC3039628

[5] Brigliadori G, Foca F, Dall’Agata M, Rengucci C, Melegari E, Cerasoli S, Amadori D, Calistri D, Faedi M (2016) Defining the cutoff value of MGMT gene promoter methylation and its predictive capacity in glioblastoma. J Neurooncol 128:333–339. 10.1007/s11060-016-2116-y 10.1007/s11060-016-2116-y 27029617

[6] Chan H-P, Samala RK, Hadjiiski LM, Zhou C (2020) Deep learning in medical image analysis, pp 3–21 10.1007/978-3-030-33128-3_1 PMC7442218 32030660

[7] Chen R, Smith-Cohn M, Cohen AL, Colman H (2017) Glioma subclassifications and their clinical significance. Neurotherapeutics 14:284–297. 10.1007/s13311-017-0519-x 28281173 10.1007/s13311-017-0519-x PMC5398991

[8] Chen H, Li C, Zheng L, Lu W, Li Y, Wei Q (2021) A machine learning-based survival prediction model of high grade glioma by integration of clinical and dose-volume histogram parameters. Cancer Med 10:2774–2786. 10.1002/cam4.3838 33760360 10.1002/cam4.3838 PMC8026951

[9] Choi RY, Coyner AS, Kalpathy-Cramer J, Chiang MF, Campbell JP (2020) Introduction to machine learning, neural networks, and deep learning. Transl Vis Sci Technol 9:14. 10.1167/tvst.9.2.14 32704420 10.1167/tvst.9.2.14 PMC7347027

[10] Deo RC (2015) Machine learning in medicine. Circulation 132:1920–1930. 10.1161/CIRCULATIONAHA.115.001593 26572668 10.1161/CIRCULATIONAHA.115.001593 PMC5831252

[11] Gilbert MR, Wang M, Aldape KD, Stupp R, Hegi ME, Jaeckle KA, Armstrong TS, Wefel JS, Won M, Blumenthal DT et al (2013) Dose-dense temozolomide for newly diagnosed glioblastoma: a randomized phase III clinical trial. J Clin Oncol 31:4085–4091. 10.1200/JCO.2013.49.6968 24101040 10.1200/JCO.2013.49.6968 PMC3816958

[12] Gittleman H, Lim D, Kattan MW, Chakravarti A, Gilbert MR, Lassman AB, Lo SS, MacHtay M, Sloan AE, Sulman EP et al (2017) An independently validated nomogram for individualized estimation of survival among patients with newly diagnosed glioblastoma: NRG oncology RTOG 0525 and 0825. Neuro Oncol 19:669–677 28453749 10.1093/neuonc/now208 PMC5464437

[13] Häni L, Kopcic M, Branca M, Schütz A, Murek M, Söll N, Vassella E, Raabe A, Hewer E, Schucht P (2022) Quantitative analysis of the MGMT methylation status of glioblastomas in light of the 2021 WHO Classification. Cancers (Basel) 14. 10.3390/cancers14133149 10.3390/cancers14133149 PMC9264886 35804921

[14] Hegi ME, Diserens A-C, Gorlia T, Hamou M-F, de Tribolet N, Weller M, Kros JM, Hainfellner JA, Mason W, Mariani L et al (2005) MGMT gene silencing and benefit from temozolomide in glioblastoma. N Engl J Med 352:997–1003. 10.1056/NEJMoa043331 15758010 10.1056/NEJMoa043331

[15] Hegi ME, Genbrugge E, Gorlia T, Stupp R, Gilbert MR, Chinot OL, Nabors LB, Jones G, Van Criekinge W, Straub J et al (2019) MGMT promoter methylation cutoff with safety margin for selecting glioblastoma patients into trials omitting temozolomide: a pooled analysis of four clinical trials. Clin Cancer Res 25:1809–1816. 10.1158/1078-0432.CCR-18-3181 30514777 10.1158/1078-0432.CCR-18-3181 PMC8127866

[16] Higa N, Akahane T, Yokoyama S, Yonezawa H, Uchida H, Takajo T, Otsuji R, Hamada T, Matsuo K, Kirishima M et al (2022) Prognostic impact of PDGFRA gain/amplification and MGMT promoter methylation status in patients with IDH wild-type glioblastoma. Neurooncol Adv 4:vdac097. 10.1093/noajnl/vdac097 10.1093/noajnl/vdac097 PMC9332894 35911637

[17] Jovčevska I (2020) Next generation sequencing and machine learning technologies are painting the epigenetic portrait of glioblastoma. Front Oncol 10 10.3389/fonc.2020.00798 PMC7243123 32500035

[18] Keime-Guibert F, Chinot O, Taillandier L, Cartalat-Carel S, Frenay M, Kantor G, Guillamo J-S, Jadaud E, Colin P, Bondiau P-Y et al (2007) Radiotherapy for glioblastoma in the elderly. N Engl J Med 356:1527–1535. 10.1056/NEJMoa065901 17429084 10.1056/NEJMoa065901

[19] Kernbach JM, Staartjes VE (2022) Foundations of machine learning-based clinical prediction modeling: part II-generalization and overfitting. Acta Neurochir Suppl 134:15–21. 10.1007/978-3-030-85292-4_3 34862523 10.1007/978-3-030-85292-4_3

[20] Kim Y, Kim KH, Park J, Yoon HI, Sung W (2023) Prognosis prediction for glioblastoma multiforme patients using machine learning approaches: development of the clinically applicable model. Radiother Oncol 183:109617. 10.1016/j.radonc.2023.109617 36921767 10.1016/j.radonc.2023.109617

[21] Lamichhane B, Daniel AGS, Lee JJ, Marcus DS, Shimony JS, Leuthardt EC (2021) Machine learning analytics of resting-state functional connectivity predicts survival outcomes of glioblastoma multiforme patients. Front Neurol 12. 10.3389/fneur.2021.642241 10.3389/fneur.2021.642241 PMC7937731 33692747

[22] Lassman A, Dimino C, Mansukhani M, Murty V, Ansell PJ, Bain E, Holen KD, Roberts-Rapp LA, Lee J, Curran W et al (2017) ACTR-68. Concordance of EGFR and MGMT analyses between local and central laboratories: implications for clinical trial design and precision medicine for depatuxizumab-mafodotin (ABT-414) in glioblastoma (GBM). Neuro Oncol 19:vi15–vi15. 10.1093/neuonc/nox168.055

[23] Leenings R, Winter NR, Plagwitz L, Holstein V, Ernsting J, Steenweg J, Gebker J, Sarink K, Emden D, Grotegerd D et al (2020) PHOTONAI -- A python API for rapid machine learning model development. 10.1371/journal.pone.0254062 10.1371/journal.pone.0254062 PMC8294542 34288935

[24] Leenings R, Winter NR, Plagwitz L, Holstein V, Ernsting J, Sarink K, Fisch L, Steenweg J, Kleine-Vennekate L, Gebker J et al (2021) PHOTONAI—A Python API for rapid machine learning model development. PLoS ONE 16:e0254062. 10.1371/journal.pone.0254062 34288935 10.1371/journal.pone.0254062 PMC8294542

[25] Li M, Dong G, Zhang W, Ren X, Jiang H, Yang C, Zhao X, Zhu Q, Li M, Chen H et al (2021) Combining MGMT promoter pyrosequencing and protein expression to optimize prognosis stratification in glioblastoma. Cancer Sci 112:3699–3710. 10.1111/cas.15024 34115910 10.1111/cas.15024 PMC8409410

[26] Liao X, Cai B, Tian B, Luo Y, Song W, Li Y (2019) Machine-learning based radiogenomics analysis of MRI features and metagenes in glioblastoma multiforme patients with different survival time. J Cell Mol Med 23:4375–4385. 10.1111/jcmm.14328 31001929 10.1111/jcmm.14328 PMC6533509

[27] Louis DN, Perry A, Wesseling P, Brat DJ, Cree IA, Figarella-Branger D, Hawkins C, Ng HK, Pfister SM, Reifenberger G et al (2021) The 2021 WHO classification of tumors of the central nervous system: a summary. Neuro Oncol 23:1231–1251. 10.1093/neuonc/noab106 34185076 10.1093/neuonc/noab106 PMC8328013

[28] Macyszyn L, Akbari H, Pisapia JM, Da X, Attiah M, Pigrish V, Bi Y, Pal S, Davuluri RV, Roccograndi L et al (2016) Imaging patterns predict patient survival and molecular subtype in glioblastoma via machine learning techniques. Neuro Oncol 18:417–425. 10.1093/neuonc/nov127 26188015 10.1093/neuonc/nov127 PMC4767233

[29] Mansouri A, Hachem LD, Mansouri S, Nassiri F, Laperriere NJ, Xia D, Lindeman NI, Wen PY, Chakravarti A, Mehta MP et al (2019) MGMT promoter methylation status testing to guide therapy for glioblastoma: refining the approach based on emerging evidence and current challenges. Neuro Oncol 21:167–178. 10.1093/neuonc/noy132 30189035 10.1093/neuonc/noy132 PMC6374759

[30] Omuro A, DeAngelis LM (2013) Glioblastoma and other malignant gliomas: a clinical review. JAMA 310:1842–1850. 10.1001/jama.2013.280319 24193082 10.1001/jama.2013.280319

[31] Ostrom QT, Bauchet L, Davis FG, Deltour I, Fisher JL, Langer CE, Pekmezci M, Schwartzbaum JA, Turner MC, Walsh KM et al (2014) The epidemiology of glioma in adults: a “state of the science” review. Neuro Oncol 16:896–913. 10.1093/neuonc/nou087 24842956 10.1093/neuonc/nou087 PMC4057143

[32] Ostrom QT, Patil N, Cioffi G, Waite K, Kruchko C, Barnholtz-Sloan JS (2020) CBTRUS statistical report: primary brain and other central nervous system tumors diagnosed in the United States in 2013–2017. Neuro Oncol 22:iv1–iv96, 10.1093/neuonc/noaa200 10.1093/neuonc/noaa200 PMC7596247 33123732

[33] Pasquini L, Napolitano A, Lucignani M, Tagliente E, Dellepiane F, Rossi-Espagnet MC, Ritrovato M, Vidiri A, Villani V, Ranazzi G et al (2021) AI and high-grade glioma for diagnosis and outcome prediction: do all machine learning models perform equally well? Front Oncol 11. 10.3389/fonc.2021.601425 10.3389/fonc.2021.601425 PMC8649764 34888226

[34] Peeken JC, Goldberg T, Pyka T, Bernhofer M, Wiestler B, Kessel KA, Tafti PD, Nüsslin F, Braun AE, Zimmer C et al (2019) Combining multimodal imaging and treatment features improves machine learning-based prognostic assessment in patients with glioblastoma multiforme. Cancer Med 8:128–136. 10.1002/cam4.1908 30561851 10.1002/cam4.1908 PMC6346243

[35] Perry A, Wesseling P (2016) Histologic classification of gliomas. Handb Clin Neurol 134:71–95. 10.1016/B978-0-12-802997-8.00005-0 26948349 10.1016/B978-0-12-802997-8.00005-0

[36] Qiu X, Gao J, Yang J, Hu J, Hu W, Kong L, Lu JJ (2020) A comparison study of machine learning (random survival forest) and classic statistic (cox proportional hazards) for predicting progression in high-grade glioma after proton and carbon ion radiotherapy. Front Oncol 10. 10.3389/fonc.2020.551420 10.3389/fonc.2020.551420 PMC7662123 33194609

[37] Sakumi K, Shiraishi A, Shimizu S, Tsuzuki T, Ishikawa T, Sekiguchi M (1997) Methylnitrosourea-induced tumorigenesis in MGMT gene knockout mice. Cancer Res 57:2415–2418 9192819

[38] Schwartzbaum JA, Fisher JL, Aldape KD, Wrensch M (2006) Epidemiology and molecular pathology of glioma. Nat Clin Pract Neurol 2:494–503; quiz 1 p following 516, 10.1038/ncpneuro0289 10.1038/ncpneuro0289 16932614

[39] Senders JT, Staples P, Mehrtash A, Cote DJ, Taphoorn MJB, Reardon DA, Gormley WB, Smith TR, Broekman ML, Arnaout O (2020) An online calculator for the prediction of survival in glioblastoma patients using classical statistics and machine learning. Clin Neurosurg 86:E184–E192. 10.1093/neuros/nyz403 10.1093/neuros/nyz403 PMC7061165 31586211

[40] Stupp R, Mason WP, van den Bent MJ, Weller M, Fisher B, Taphoorn MJB, Belanger K, Brandes AA, Marosi C, Bogdahn U et al (2005) Radiotherapy plus concomitant and adjuvant temozolomide for glioblastoma. N Engl J Med 352:987–996. 10.1056/NEJMoa043330 15758009 10.1056/NEJMoa043330

[41] Valdebenito J, Medina F (2019) Machine learning approaches to study glioblastoma: a review of the last decade of applications. Cancer Rep 2. 10.1002/cnr2.1226 10.1002/cnr2.1226 PMC7941469 32729254

[42] Wang L, Li Z, Liu C, Chen L, Liu L, Hu Z, Zhao L, Lu D, Teng L (2017) Comparative assessment of three methods to analyze MGMT methylation status in a series of 350 gliomas and gangliogliomas. Pathol Res Pract 213:1489–1493. 10.1016/j.prp.2017.10.007 29103769 10.1016/j.prp.2017.10.007

[43] Weller M, van den Bent M, Preusser M, Le Rhun E, Tonn JC, Minniti G, Bendszus M, Balana C, Chinot O, Dirven L et al (2021) EANO Guidelines on the diagnosis and treatment of diffuse gliomas of adulthood. Nat Rev Clin Oncol 18:170–186. 10.1038/s41571-020-00447-z 33293629 10.1038/s41571-020-00447-z PMC7904519

[44] Wick W, Platten M, Meisner C, Felsberg J, Tabatabai G, Simon M, Nikkhah G, Papsdorf K, Steinbach JP, Sabel M et al (2012) Temozolomide chemotherapy alone versus radiotherapy alone for malignant astrocytoma in the elderly: the NOA-08 randomised, phase 3 trial. Lancet Oncol 13:707–715. 10.1016/S1470-2045(12)70164-X 22578793 10.1016/S1470-2045(12)70164-X

[45] Wick W, Weller M, van den Bent M, Sanson M, Weiler M, von Deimling A, Plass C, Hegi M, Platten M, Reifenberger G (2014) MGMT testing–the challenges for biomarker-based glioma treatment. Nat Rev Neurol 10:372–385. 10.1038/nrneurol.2014.100 24912512 10.1038/nrneurol.2014.100

[46] Yuan G, Niu L, Zhang Y, Wang X, Ma K, Yin H, Dai J, Zhou W, Pan Y (2017) Defining optimal cutoff value of MGMT promoter methylation by ROC analysis for clinical setting in glioblastoma patients. J Neurooncol 133:193–201. 10.1007/s11060-017-2433-9 28516344 10.1007/s11060-017-2433-9

[47] Zhu Q, Liang Y, Fan Z, Liu Y, Zhou C, Zhang H, He L, Li T, Yang J, Zhou Y et al (2022) Development and validation of a novel survival prediction model for newly diagnosed lower-grade gliomas. Neurosurg Focus 52:E13. 10.3171/2022.1.FOCUS21596 35364578 10.3171/2022.1.FOCUS21596

